# Large manipulative experiments revealed variations of insect abundance and trophic levels in response to the cumulative effects of sheep grazing

**DOI:** 10.1038/s41598-017-11891-w

**Published:** 2017-09-12

**Authors:** Jingchuan Ma, Xunbing Huang, Xinghu Qin, Yong Ding, Jun Hong, Guilin Du, Xinyi Li, Wenyuan Gao, Zhuoran Zhang, Guangjun Wang, Ning Wang, Zehua Zhang

**Affiliations:** 1 State Key Laboratory for Biology of Plant Diseases and Insect Pests, Institute of Plant Protection, Chinese Academy of Agricultural Sciences, Beijing, 100193 P.R. China; 2 Scientific Observation and Experimental Station of Pests in Xilingol Rangeland, Ministry of Agriculture, Institute of Plant Protection, Chinese Academy of Agricultural Sciences, Xilinhot, 02600 P.R. China; 3 Scottish Oceans Institute, University of St Andrews, East Sands, St Andrews, KY16 8LB Scotland UK; 4 Institute of Grassland Research, Chinese Academy of Agricultural Sciences, Hohhot, 010010 P.R. China; 5 National Animal Husbandry Service, Ministry of Agriculture, Beijing, 100125 P.R. China; 6 Grassland Workstation, Inner Mongolia, Hohhot, 010020 P.R. China

## Abstract

Livestock grazing can affect insects by altering habitat quality; however, the effects of grazing years and intensities on insect abundance and trophic level during manipulative sheep grazing are not well understood. Therefore, we investigated these effects in a large manipulative experiment from 2014 to 2016 in the eastern Eurasian steppe, China. Insect abundance decreased as sheep grazing intensities increased, with a significant cumulative effect occurring during grazing years. The largest families, Acrididae and Cicadellidae, were susceptible to sheep grazing, but Formicidae was tolerant. Trophic primary and secondary consumer insects were negatively impacted by increased grazing intensities, while secondary consumers were limited by the decreased primary consumers. Poor vegetation conditions caused by heavy sheep grazing were detrimental to the existence of Acrididae, Cicadellidae, primary and secondary consumer insects, but were beneficial to Formicidae. This study revealed variations in insect abundance and trophic level in response to continuous sheep grazing in steppe grasslands. Overall, our results indicate that continuous years of heavy- and over- sheep grazing should be eliminated. Moreover, our findings highlight the importance of more flexible sheep grazing management and will be useful for developing guidelines to optimize livestock production while maintaining species diversity and ecosystem health.

## Introduction

Excessive grazing on grassland ecosystems by livestock poses a serious threat to the grasslands by lowering productivity, biodiversity and stability^1^, leading to ecological deterioration^2^, especially for insect diversity^3^. Insects are a major, but often under-appreciated component of terrestrial ecosystems^4–6^, and the effects of grazing on insect diversity have been thoroughly investigated in previous studies. Some studies have shown that grazing may increase insect diversity^7–9^, while others showed decreased insect diversity^10, 11^ or no change in insect diversity^12, 13^ in response to grazing. These inconsistent results may be due to factors such as variation in vegetation^6^, intensity of grazing^14, 15^, and herbivore size^6^. However, growing evidence shows that insects are experiencing local/regional species loss or even global extinction^16^, and that the diversity of insects apparently declines more rapidly than that of vertebrates and plants^6, 17^. Therefore, understanding critical factors that determine their diversity and species composition has become an urgent task facing ecologists and conservation biologists.

The effects of large herbivores on insect abundance are grazer species-specific and pre-grazing plant diversity-dependent^9, 18^. Large herbivore species can alter vegetation features due to diet selection and body size, potentially influencing the insect community^13, 19^. Low grazing intensities result in taller swards, providing more forage and shelter for herbivorous insects^20^. Grazing profoundly changes insect taxonomic composition^6^. Although extensive research investigating the effects of livestock grazing on grassland insect abundance has provided valuable insights^21, 22^, several important gaps in our knowledge remain. Previous studies investigating the effects of grazing intensities on insect diversity and abundance often only compared two or three stocking rates^13, 23, 24^, and continuous grazing years have rarely been considered. Indeed, some studies only provided one year of data, or conducted short term investigation of grazing at few levels of grazing intensity^25, 26^ that lacked continuous treatments^6, 19^. Moreover, other studies only focused on limited groups, such as pollinators^24^. The use of taxonomic hierarchies such as order and species levels could be advantageous to biodiversity assessments^27^. Thus, there is a pressing need for studies that examine how the taxonomic compositions of insect communities respond to livestock grazing. Furthermore, the effects of continuous manipulative grazing on other insect groups, such as Formicidae, Acrididae, Cicadellidae, and on various trophic levels, are not well understood. Such knowledge will facilitate reasonable grazing management and maintenance of species diversity and ecosystem health.

Grasslands comprise the largest terrestrial ecosystem in China, and play a critical role in maintaining the structure, function and stabilization of surrounding natural ecosystems^28, 29^. Sheep grazing is a key management tool in the steppe grassland of Northern China and the most important economic income source of herdsman^30^. However, rapid steppe degradation has led to reduced biodiversity, decreased productivity and, in some cases, desertification owing to livestock over-grazing^31, 32^. The effects of continuous sheep grazing on insect abundance and trophic level in such areas are unknown; therefore, we conducted a 3-year large manipulative experiment with five grazing levels (0, 4, 8, 12 and 16 sheep per 1.33 ha) in Inner Mongolia, China. The specific goals of this study were to determine (i) how dominant insect groups respond to various intensities and years of sheep grazing; (ii) how insect trophic levels change with sheep grazing; and (iii) potential reasons for insect abundance variation for sheep grazing. Suggestions regarding how to manage sheep grazing to maintain insect diversity are also discussed.

## Results

### Insect Shannon-Wiener index, species richness, and abundance

**Figure 1:**
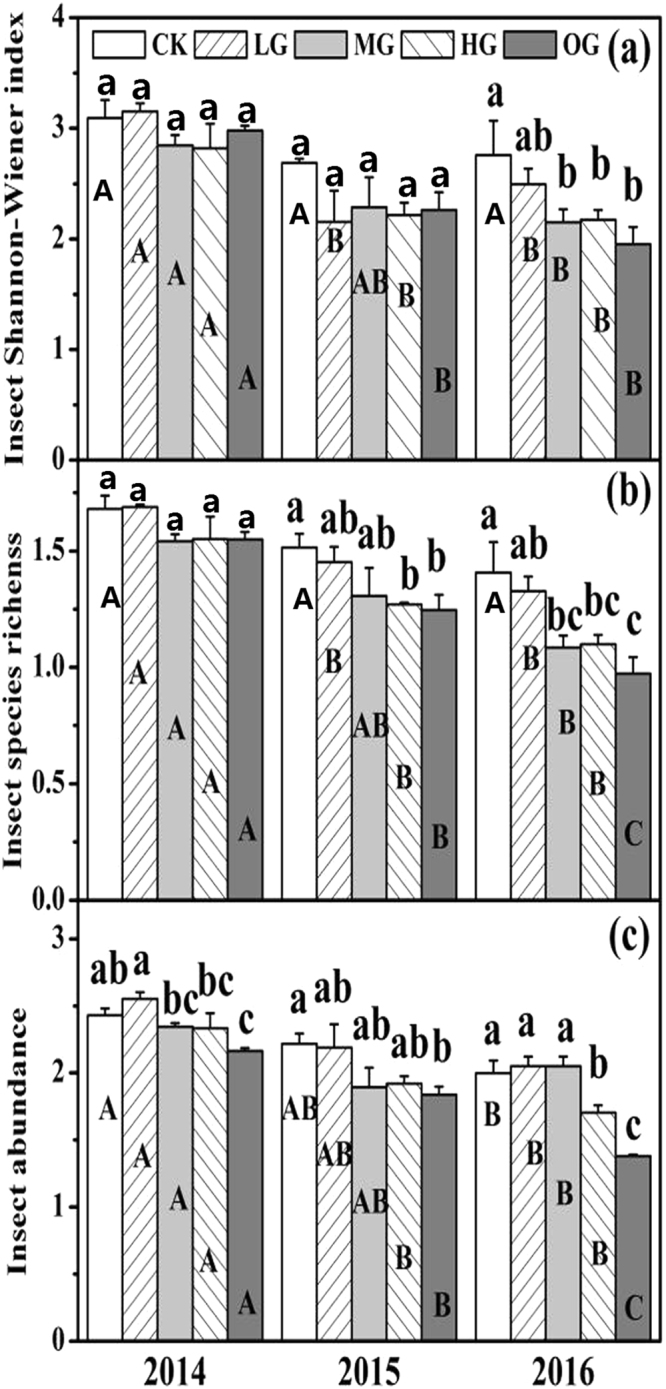
Effect of sheep grazing intensity and grazing years on insect Shannon-Wiener index (**a**), species richness (**b**), and abundance (**c**). Values represent means ± SE. Different lowercase letters above the bars indicate that values differ significantly between altered grazing intensities treatments within each experimental year at *P* < 0.05. Different capital letters indicate that values differ significantly between altered grazing years within each grazing intensity at *P* < 0.05.

Grazing intensities and grazing years were found to have significant effects on the insect Shannon-Wiener index, species richness, and abundance (all *P* < 0.05, Table 1). When compared with the control, moderate grazing (MG), heavy grazing (HG) and over grazing (OG) led to a significant (*P* < 0.05) reduction in the insect Shannon-Wiener index in 2016 (Fig. 1a). Moreover, insect species richness decreased significantly (*P* < 0.05) in 2015 and 2016 in response to grazing treatments MG, HG and OG (Fig. 1b). Furthermore, insect abundance was significantly lower (*P* < 0.05) in response to grazing treatments HG and OG in all three experimental years (Fig. 1c). These results indicate that grazing intensity can significantly influence insect variation.

**Table 1:** Results of repeated-measures ANOVA of the effects of grazing years (Y) and grazing intensities (G) and their interactions on insect variables.

Insect variables		Y	G	Y*G
		2, 30	4, 30	8, 30
Insect Shannon-Wiener index	F	23.83***	3.6*	0.91 NS
Insect species richness	F	47.55***	9.61***	0.72 NS
Insect abundance	F	51.64***	15.14***	2.19 NS
Formicidae abundance	F	13.96***	0.31 NS	3.66**
Acrididae abundance	F	53.21***	7.79**	1.93 NS
Cicadellidae abundance	F	32.62***	15.57***	0.77 NS
Primary consumer abundance	F	34.82***	8.49***	0.65 NS
Secondary consumer abundance	F	10.54***	7.32***	2.27*

including insect Shannon-Wiener index, insect species richness, insect abundance, Formicidae abundance, Acrididae abundance, Cicadellidae abundance, primary consumer abundance and secondary consumer abundance. Significance: ***P < 0.001; **P < 0.01; *P < 0.05; NS: not significant (*P* > 0.05).

There were significant inter-annual changes in three insect variables (Fig. 1). When compared with 2014, continuous sheep grazing in 2015 and 2016 led to a significant (*P* < 0.05) reduction in the insect Shannon-Wiener index, species richness, and abundance, especially for over grazing (OG). These results showed that sheep grazing had a significant negative cumulative effect on insects.

### Formicidae, Acrididae, and Cicadellidae abundance

Grazing intensities and years had significant effects on the largest families, Formicidae, Acrididae, and Cicadellidae (all *P* < 0.05, Table 1), as well as a significant interactive effect on Formicidae (*P* < 0.05). Acrididae abundance exhibited a significant negative linear relationship to grazing intensities in 2016 (y = −0.642x + 14.867, R^2^ = 0.33, *P* = 0.01425). Cicadellidae abundance also showed significant negative linear relationships to grazing intensities in all three experimental years (2014: y = −0.642x + 14.867, R^2^ = 0.33, *P* = 0.01425; 2015: y = −2.9x + 78, R^2^ = 0.312, *P* = 0.0178; 2016: y = −2.25x + 43.8, R^2^ = 0.33, *P* = 0.0004). In contrast, there were no significant changes between Formicidae abundance and grazing intensities in any of the experimental years. These results suggest that the dominant insect groups responded differently to altered sheep grazing intensities.

**Figure 2:**
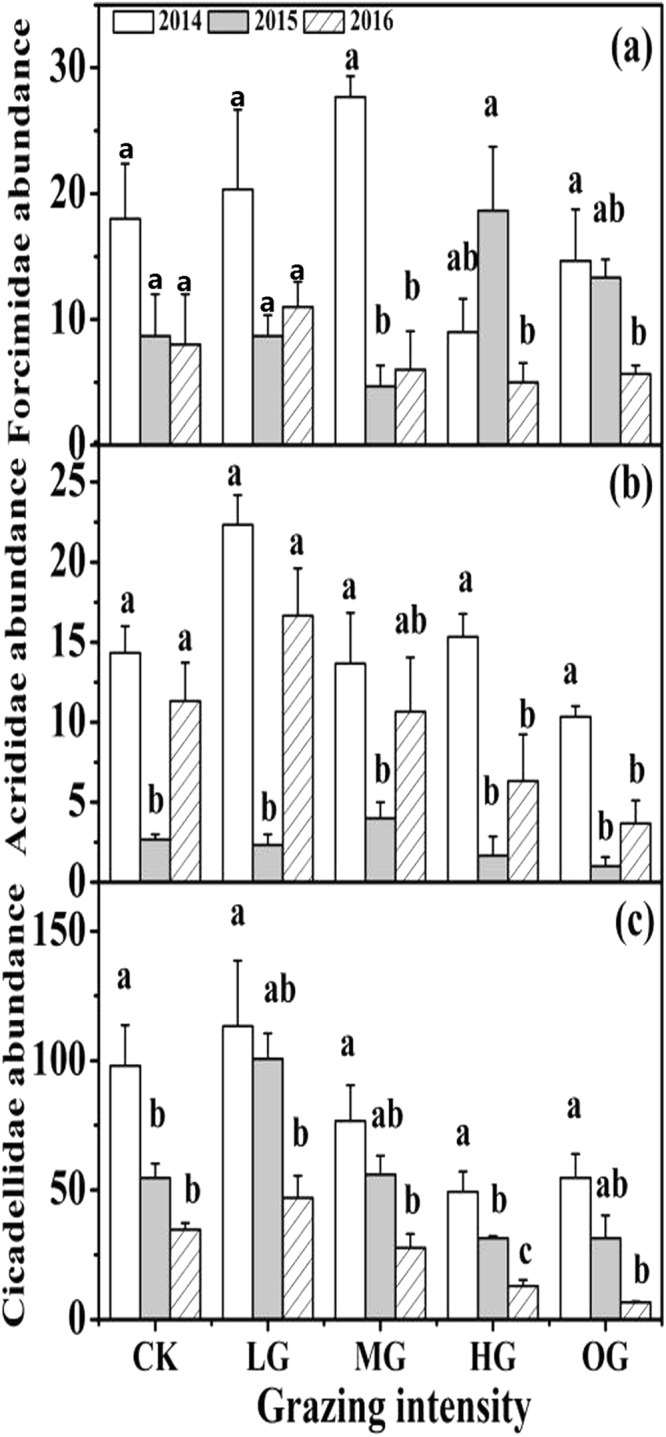
Effect of grazing years on Formicidae, Acrididae and Cicadellidae. Values represent the means ± SE. Different lowercase letters above the bars indicate that values differed significantly between altered grazing years within each grazing intensity at *P* < 0.05.

There were also significant inter-annual changes in these three dominant insect groups (Fig. 2). When compared with 2014, continuous heavy grazing (HG) and over grazing (OG) by sheep in 2015 and 2016 led to a significant (*P* < 0.05) reduction in the abundance of Formicidae, Acrididae, and Cicadellidae, which suggested a significant negative cumulative effect on grazing years.

### Primary and secondary consumers

Grazing intensity and years all significantly influenced primary consumers (phytophagous insects) and secondary consumers (parasitoids and carnivorous insects) (all *P* < 0.05, Table 1), and these factors exerted a significant interaction effect on secondary consumers (*P* < 0.05). Additionally, both primary and secondary consumers showed a significant negative linear relationship with grazing intensity (primary consumer, 2014: y = 246.933 − 6.858x, R^2^ = 0.262, P = 0.030, 2015: y = 147.877 − 6.767x, R^2^ = 0.309, P = 0.018, 2016: y = 91.8 − 4.667x, R^2^ = 0.600, P = 0.0004; secondary consumer, 2015: y = 22.067 − 1.05x, R^2^ = 0.415, P = 0.0057, 2016: y = 15.333 − 0.667x, R^2^ = 0.268, P = 0.028). These results indicate that both primary and secondary consumers were also susceptible to altered sheep grazing intensities.

**Figure 3:**
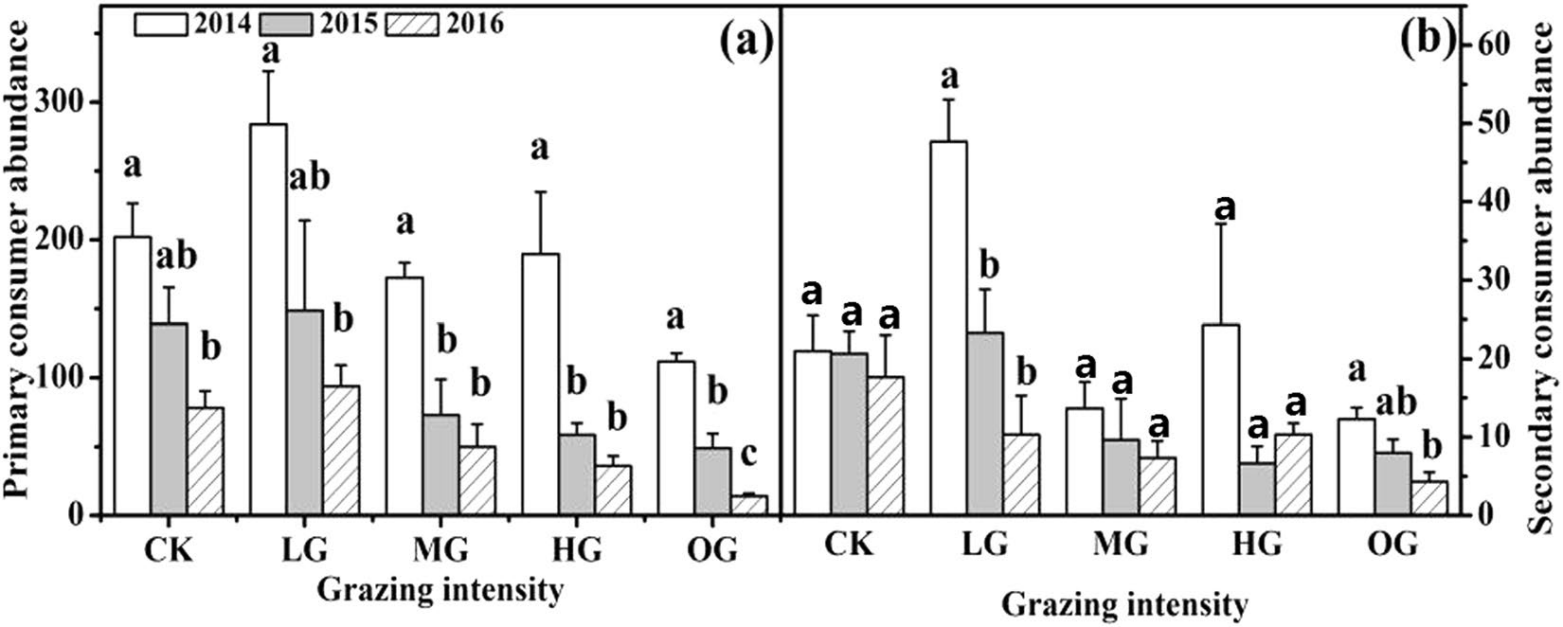
Effect of grazing years on primary consumers, secondary consumers. Values represent the means ± SE. Different lowercase letters above the bars indicate that values differ significantly between altered grazing years within each grazing intensity at *P* < 0.05.

There were also significant inter-annual changes (Fig. 3). Compared with 2014, the continuous sheep grazing in 2015 and 2016 led to a significant (*P* < 0.05) reduction on these two insect trophic levels, especially for over grazing (OG). These results showed that there also had a significant negative cumulative effect of sheep grazing years on primary consumer and secondary consumer.

**Figure 4:**
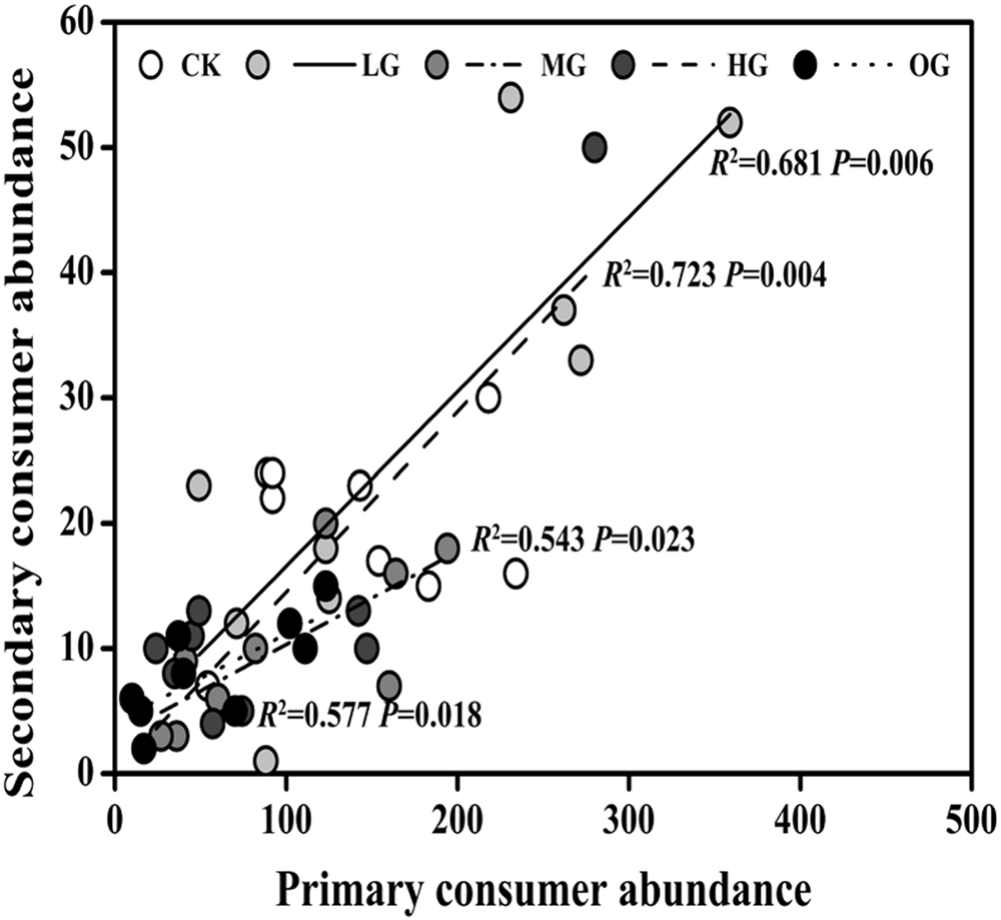
Relationship between primary consumer (phytophagous) abundance and secondary consumer (parasitoids and predators) abundance. The solid line represents their relationship under light grazing (LG, R^2^ = 0.681, *P* = 0.006), the dashed line represents their relationship under moderate grazing (MG, R^2^ = 0.723, *P* = 0.004), the dashed dotted line represents their relationship under heavy grazing (HG, R^2^ = 0.543, *P* = 0.023), and the dotted line represents their relationship under over grazing (OG, R^2^ = 0.577, *P* = 0.018). Each data point represents the value in one plot for each grazing intensity across the three experimental years (2014 to 2016).

The relationship between primary and secondary consumer abundance were examined. The result showed that the secondary consumer abundance significantly (*P* < 0.05) increased with increasing primary consumer abundance in all grazing treatments (Fig. 4). These findings suggest that the secondary consumer insects were limited by decreased primary consumer insects following increased sheep grazing intensities.

### Grazing-vegetation-insect relationship

The Importance Value showed that the plant *Leymus chinensis* (Trin.) Tzvel. is the most widely distributed across each grazing plot, followed by *Stipa grandis* P. Smirn., *Cleistogenes squarrosa* (Trin.) Keng., *Chenopodium glaucum* L. and *Carex korshinskyi* Kom. (Table S1). There were no significant differences in plant diversity and species richness among grazing intensities in 2015 (Fig. S1). The relationship between vegetation structure heterogeneity and grazing intensities was an approximate U-curve (Fig. S1) with a tendency to first ascend, then descend.

**Figure 5:**
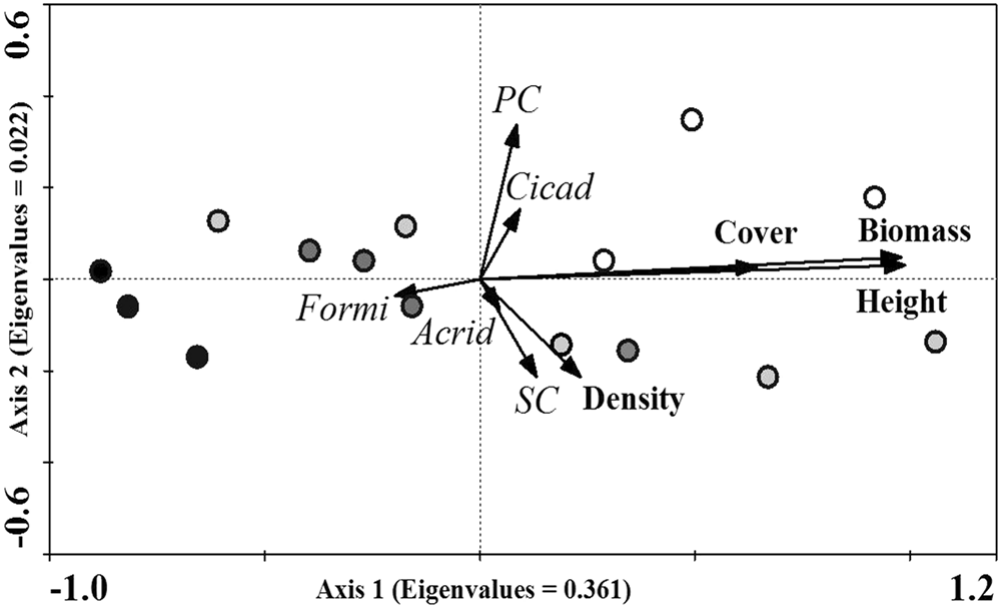
Redundancy analysis (RDA) of the response of insect abundance to changes in vegetation in response to various grazing intensities. Open circles represent the plant immunity status for the control treatment (CK). Light gray symbols represent the plant immunity status for light grazing treatment (LG). Gray symbols represent the plant immunity status for moderate grazing (MG) treatment. Dark gray symbols represent the plant immunity status for heavy grazing (HG) treatment. Dark symbols represent the plant immunity status for over grazing (OG) treatment.

The grazing-vegetation-insect relationships in 2015 were next examined by redundancy analysis (RDA) (Fig. 5). Axis 1 explained most of the variation in insect abundance (37.3%). The results showed that the grasslands subjected to heavy grazing (HG) and over grazing (OG) were characterized by decreased plant coverage, biomass, density and height (Fig. 5, Table S2). In contrast, grasslands subjected to no grazing (CK), light grazing (LG), and moderate grazing (MG) showed high plant coverage, biomass, density and height (Fig. 5, Table S2). These four vegetation variables showed positive relationships with Acrididae, Cicadellidae, primary consumer and secondary consumer insect abundance (Fig. 5, Table S3). Moreover, the results showed that the poorer vegetation attributes of the HG and OG groups could be detrimental to the existence of Acrididae, Cicadellidae, primary consumer and secondary consumer insects, but would be beneficial to Formicidae. These results suggest that the changes in vegetation in response to different grazing intensities resulted in variations in the insect community and explained these changes well.

## Discussion

Understanding the relationship between grazing and biodiversity and how it affects insect populations may provide insights that improve monitoring strategies, early warning alerts and management strategies for species conservation^22, 33^. In this study, the cumulative effects of sheep grazing intensities and years on insect abundance and trophic level were analyzed by a large manipulative experiment over three years. Several broad conclusions can be drawn based on the results. First, sheep grazing intensities significantly influenced the insect community, with increased grazing having detrimental effects on insect abundance, diversity and species richness. These findings confirm those of previous studies linking changes in insect population dynamics to variations in grazing intensity^3, 9, 25, 34^. Second, sheep grazing years also negatively affected the insect community with a significant cumulative effect. This finding agrees with those of previous studies showing that continuous grazing years were detrimental to insect abundance^35^, especially under heavy grazing and over grazing. Third, we found the largest families, Acrididae and Cicadellidae, were susceptible to sheep grazing intensity, but Formicidae was not. This result also supports the general opinion that insect groups respond to grazing differently^6^, and that not all species are sensitive to grazing^27^. Fourth, grazing can affect insect trophic level. Interestingly, we found that primary and secondary consumers were both negatively impacted by increasing grazing intensity, with the secondary consumer being limited by the primary consumer. These results were contrary to those of previous studies that indicated extensive grazing enhanced phytodiversity^36, 37^, and in turn promoted species richness of higher trophic levels^38^. This indicates that there is a cascade reaction to insects in response to grazing. Alterations in one or some insect species usually change other the specific interspecies relationships of other insect species^39^. Finally, we found that poorer vegetation attributes associated with increased sheep grazing intensity were detrimental to insect existence, and that plant attributes for grazing were closely associated with variations in insect abundance^3, 6, 9^.

The question of which selective factors for grazing have driven the insect change is of great interest, and has been investigated in many previous studies^1, 6, 40, 41^. Livestock grazing can affect insect diversity and abundance directly and indirectly. The direct effects include unintentional ingestion or trampling^42^, while the indirect effects mainly include changes in microclimates, vegetation, and interspecies relationships in response to grazing^14, 40^. As human disturbances of native grasslands increase, understanding the grazing-plant-insect relationship becomes increasingly important^6, 18^. Grazing and trampling can modify vegetation, thereby altering the insect community structure^43, 44^. Large herbivores may also lower the quality of plants by reducing their nitrogen levels^22^ and change vegetation structure by exposing bare soil; thus, increasing the risk of predators^45^. Foraging by livestock reduces plant density and coverage, reducing food availability to insects. Herbivorous insects normally need adequate food resources to support their development and reproduction^46^. When food resources decrease and become scarce, their population dynamics are also be negatively affected by food shortages^47^. Therefore, there is generally a positive relationship between herbivorous insects and plant biomass, especially during grazing of degraded grasslands of limited resources^48, 49^. Since insects primarily feed on plants for survival^19, 20^, plant diversity has enhances insect diversity and abundance^21–23^. Accordingly, decreased plant coverage, height, biomass and plant litter can be presumed to lead to decreased quality of habitat through exhausted food resources, shifts in the nutrient status of host plants and development of inhospitable microclimates^17^. For example, fruit flies inhabit grasslands^50^ when plant coverage is high, but areas of exposed bare soil do not attract colonizing adults^51^. Zhu *et al*.^19^, showed that large herbivores strongly affected insect species richness by modifying plant structural heterogeneity, which reversed the positive relationship between plant and insect diversity. Zhong *et al*.^9^, found that the positive interactions between large herbivores and grasshoppers were driven by differential herbivore foraging preferences for plant resources that break down the associational plant defense between grasses and forbs. In this study, we found that vegetation structure heterogeneity tended to ascending, then descending in succession in response to increased sheep grazing intensity, which supported the intermediate disturbance hypothesis^52^. Plant coverage, biomass, density and height were all tightly correlated with insect abundance. Specifically, decreased vegetation variables in response to increased grazing intensity deteriorated insect food resources and refuge, which resulted in decreased insect abundance, especially for phytophagous insects (the primary consumer), such as the dominant groups Acrididae and Cicadellidae. As a result, secondary consumers suffered from the decreased food availability owing to the shortage of primary consumers. Interestingly, we found that different grazing intensities did not influence plant biodiversity and species richness, while insect diversity and abundance decreased with increased grazing intensity in 2015. This finding strongly supports a recent report that showed arthropod diversity is often more negatively affected by grazing than plant diversity^40^. Furthermore, the microclimate variables in different grazing habitats, such as humidity, light, and temperature, can also influence insect growth performance and population dynamics^53^. These variables may be more unfavorable to insects following increased grazing intensity. Thus, future research should be focus on differences in micro-climate variables and how they influence insect abundance for sheep grazing.

We also found that yearly fluctuations of grazing would impact on insect abundance more than grazing treatment. One reason is the cumulative effects of continuous sheep grazing, that have been demonstrated by Minckley *et al*.^35^ and Marriott *et al*.^36^ and in our present study. Another reason may be climatic variation of different grazing years, such as the temperature and rainfall. Stige *et al* ^54^. have demonstrated that climatic changes of yearly fluctuation could significantly impact insect population dynamics. In addition, insect migration and short distance dispersal in response to habitat change within years may also impact on insect abundance variation^45^. Those related researches can well explain grazing years’ effect on insect abundance.

Insects are important indicators of the ecological environment^54^. Monitoring insect communities to measure the benefits of changes in grazing intensities to improve biodiversity should consider the continuous effects of grazing disturbance. Although Cicadellidae is one of the largest families of phytophagous insects and among the dominant groups of prairie herbivores^55^, its abundance significantly declined during three years of consecutive grazing, indicating that the insects may not be able to tolerate the habitat conditions created by this grazing regimen. Additionally, grazing has been shown to significantly reduce Acrididae, which are important grassland pests^14^. Acrididae density in 2016 differed markedly from the first year of grazing, suggesting that continuous grazing may also negatively influence their population dynamics and spatial distribution. The Acrididae and Cicadellidae were susceptible and fragile to sheep grazing. Although most insect groups decreased with decreased habitat quality, the Formicidae group did not change. The mechanism regulating this behavior is yet to be determined; however, we presume that this may have resulted from the availability of suitable food resources such as herbivore feces, plant litter and microclimate, such as adequate sunshine. These differences in insect responses indicate a difference in sensitivity for grazing based on a likely complex mechanism, which will be addressed in future studies. Nevertheless, our results showed that not all insect species are susceptible to grazing by large herbivores^6^, implying that susceptible insect species may decline or disappear and should therefore be conserved in grazed grasslands. The results of the present study also suggest that insect groups and species respond to herbivore grazing in a complex manner, and it is essential to quantify this effect in further studies.

Livestock grazing is a key management tool in grasslands, and its widespread prevalence has generated great interest in understanding its ecological effects, especially for insects^40, 56^. Against the background of decreased biodiversity^57, 58^, appropriate management of remaining grassland sites is required to maintain biodiversity. Grazing appears to have a high potential for combining these targets with the growing social demands for animal welfare^59^. However, the main function of pastures for farmers is to meet agronomic and financial interests. Therefore, identification of a threshold grazing intensity that fulfils both environmental and livestock production objectives is essential. The insights into the relationship between sheep grazing and insect abundance provide an opportunity to devise strategies to improve livestock and insect management that reduce the potential conflict of livestock production and insect conservation. It is well known that high stocking rates combined with intensive grassland management contribute to the deterioration of insect diversity^37^. However, it is still not clear what level of grazing intensity is appropriate to conserve insects and by which mechanisms sheep grazing intensity affects insect diversity in Chinese Steppe grasslands. We found that the continuous years of heavy and over grazing should be eliminated to prevent a large decrease in insect abundance. This can be accomplished through interventions such as improved livestock grazing management and implementation of fallow periods to allow habitat recovery. Moreover, grazing intensity and years should be considered to ensure good livestock management. Finally, although we studied the vegetation and insect responses to sheep grazing for three years, the observed responses to sheep grazing may only have just been beginning. Overall, a better understanding of sheep grazing ecosystems requires long-term monitoring, particularly in relation to improving grassland habitats to ensure sustainability, plant and insect biodiversity and the livelihood of farmers.

## Materials and Methods

### Study site

The study site is located in the eastern region of the Eurasian Steppe Zone, Inner Mongolia, China (116°32′E, 44°15′N) and managed by the institute of Grassland Research, Chinese Academy of Agricultural Sciences. The site has a semi-arid continental climate with a mean annual temperature of −0.1 °C, with the coldest temperatures occurring in January (average −22.0 °C, extreme minimum −41 °C) and the highest in July (average 18.3 °C, extreme maximum 38.5 °C). The annual accumulated temperature ranges from 2100 °C to 2400 °C, and the annual precipitation was 350 to 450 mm. The major soil type in the area is calcic chestnut soil and the main vegetation type is typical steppe dominated by two perennial grasses, *Leymus chinensis* (Trin.) Tzvel. and *Stipa grandis* P. Smirn. Other common grass species include *Cleistogenes squarrosa* (Trin.) Keng., *Artemisia frigida* Wild., *Salsola collina* Pall., and *Chenopodium glaucum* L. The period of maximum vegetation biomass and highest insect occurrence is from early July to early August. The experimental site was not grazed, but was mowed from 2007 to 2013.

### Experimental design and animal management

A relatively flat area within the study site with homogenous conditions of soil and plants was blocked in 2014. We constructed 15 sets of 1.33-ha enclosures with a 100 m × 125 m dimension and a 1.5 m-high iron netting above the ground to confine sheep within the enclosures. Fifteen enclosures were randomly assigned into five treatments with three replicates per treatment: control (CK), light grazing (LG), moderate grazing (MG), heavy grazing (HG) and over grazing (OG). Based on the local standard set^60^ for sheep grazing intensity, 0 (grazing pressure: 0 SSU·d·(hm^2^)^−1^·y^−1^), 4 (170 SSU·d·(hm^2^)^−1^·y^−1^), 8 (340 SSU·d·(hm^2^)^−1^·y^−1^), 12 (510SSU·d·(hm^2^)^−1^·y^−1^) and 16 (680 SSU·d·(hm^2^)^−1^·y^−1^) sheep per 1.33 ha were used for the CK, LG, MG, HG and OG groups, respectively. The grazing season lasted from June 10 to September 10 each year.

### Insect survey

Since the general records for the study area indicate that the highest insect diversity occurs in July, insect samples were collected on 2014 (July 10), 2015 (July 10) and 2016 (July 11), respectively. Insects were collected using a suction sampler as previously described^61^. To estimate the abundance of each insect species per square meter, insects were sucked from a steel framed 1 m × 1 m × 1 m quadrat randomly placed in five locations within each plot. Each surface of the quadrat frame, excluding the undersurface, was covered with fine 1 mm^2^ cloth mesh to ensure that no insects escaped. The covered upper mesh could be opened when using suction sampler. To avoid man-made disturbances and collect as many insects as possible, the quadrat frame was thrown into the air and allowed to freely fall to the ground. We then used the suction sampler to collect all insects within the quadrat, which took 2 minutes for each suction sample. These five sample locations for each plot had a relatively flat terrain with uniform vegetation and were at least 10 m away from the plot boundary to minimize edge effects. Insect specimens were only collected under favorable conditions (sunny days with minimal cloud cover and calm or no wind) from 09:00 to 15:00 h. All grazed plots were sampled in a random order. All contents of the suction sampler were preserved in ziplock bags marked with respective plot numbers and taken to the laboratory. We then killed all insects by freezing them (−20 °C for 20 hours).

The insects were examined and sorted into 10 orders; Hemiptera, Orthoptera, Hymenoptera, Coleoptera, Diptera, Lepidoptera, Neuroptera, Collembola, Thysanoptera and Mantodea. All individuals were identified to species, but immature insects omitted from analysis. Insects that could not be identified as a species were separated into recognizable taxonomic units based on morphological characteristics.

### Vegetation survey

Vegetation was sampled in parallel with insect surveys in 2015. The following vegetation attributes were evaluated in five randomly selected quadrats (1 m × 1 m) within each plot using the same methods by Zhu *et al*.^24^: plant coverage, height, density, biomass. Plant samples were not taken from about 10 m from the plot boundary to avoid any edge effects. The tufted plant (*S. grandis*) were counted by the number of tufts. The Importance Value (I.V.) of each plant species/treatment was calculated by the formula^62^: I.V. = relative cover + relative density + relative frequency (Table S1).

### Statistical analyses

Insect and plant Shannon-Weiner index^63^ was calculated as

$$H=-{\sum }_{1}^{{\rm{S}}}(Pi\times \,\mathrm{ln}\,Pi)$$

where *Pi* is the proportion of individuals represented by species *i*, *S* is the number of species and *H* is the Shannon-Weiner diversity index. Insect species richness and abundance within each plot from 2014 to 2016 were also analyzed. Vegetation structural heterogeneity was estimated as the coefficient of variation (CV) of plant height in each quadrat^19^.

Normality of these measured variables of plants and insects was assessed using SAS^64^, and insect species and abundance were log-transformed prior to analysis. We used repeated-measures ANOVA to evaluate the effects of grazing intensity and grazing years on the insect Shannon-Wiener index, insect species and insect abundance, with grazing intensity treatment as a between-subject factor (main effect) and years as a within-subject factor (repeated), considering plots as experimental units. Furthermore, one-way ANOVA with LSD post hoc tests was used to compare insect variables within each year or each intensity using SAS version 8.0. Relationships between grazing intensity and abundance of the dominant insect groups (Acrididae, Cicadellidae and Formicidae) and both trophic primary and secondary consumer insect abundance were analyzed using the General Linear Model (GLM), respectively.

Multivariate ordination redundancy analysis (RDA) allows simultaneous representation of observations, Y variables, and X variables in two or three dimensions, which is optimal for the covariance criterion^65, 66^. We evaluated the relationships between grazing intensity-vegetation attribute-insect abundance by RDA using treatment plots as observations, vegetation parameters (biomass, coverage, density, height) as environmental variables and abundance of insect groups as species variables.

## Electronic supplementary material

Supplementary information
