# Supplementary material for: Large manipulative experiments revealed variations of insect abundance and trophic levels in response to the cumulative effects of sheep grazing
Source: Sci Rep. 2017 Sep 12;7:11297. doi: 10.1038/s41598-017-11891-w (PMC5595790; doi:10.1038/s41598-017-11891-w)
Supplement: Supplementary file 1 — Supplementary information [file 41598_2017_11891_MOESM1_ESM.pdf]

Large manipulative experiments revealed variations of insect abundance and trophic levels in response to the cumulative effects of sheep grazing

Jingchuan Ma, Xunbing Huang, Xinghu Qin, Yong Ding, Jun Hong, Guilin Du, Xinyi Li, Wenyuan Gao, Zhuoran Zhang, Guangjun Wang, Ning Wang, Zehua Zhang

Table S1 Descriptions of plant community composition and plant species importance values at five grazing levels in the experimental site.

Plant species	Importance value				
	CK	LG	MG	HG	OG
Leymus chinensis	0.425	0.359	0.225	0.314	0.238
Stipa grandis	0.060	0.046	0.098	0.067	0.075
Cleistogenes squarrosa	0.075	0.078	0.100	0.179	0.058
Chenopodium glaucum	0.118	0.174	0.234	0.268	0.233
Carex korshinskyi	0.062	0.098	0.108	0.154	0.105
Salsola collina	0.016	0.044	0.051	0.079	0.071
Koeoeria cristata	0.007	0.000	0.000	0.000	0.000
Allium anisopodium	0.015	0.026	0.020	0.011	0.021
Allium bidentatum	0.006	0.000	0.000	0.000	0.010
Allium tenuissimum	0.014	0.008	0.020	0.000	0.021
Allium ramosum	0.007	0.008	0.011	0.000	0.010
Artemisia annua	0.020	0.022	0.000	0.011	0.000
Astragalus galactites	0.007	0.008	0.010	0.011	0.011
Chenopodium aristatum	0.008	0.012	0.041	0.012	0.012
Melissitus ruthenica	0.013	0.017	0.010	0.032	0.000
Iris tenuifolia	0.006	0.000	0.000	0.000	0.010
Gueldenstaedtia verna	0.007	0.000	0.000	0.000	0.000
Thalictrum aquilegifolium	0.000	0.000	0.010	0.011	0.010
Carpesium abrotanoides	0.013	0.029	0.020	0.022	0.010
Axyris amaranthoides	0.006	0.000	0.000	0.000	0.010
Heteropappus altaicus	0.008	0.001	0.000	0.000	0.000
Potentilla tanacetifolia	0.006	0.000	0.000	0.000	0.000
Artemisia sieversiana	0.000	0.017	0.000	0.000	0.010
Dontostemon	0.000	0.010	0.010	0.000	0.000
Setaria viridis	0.000	0.008	0.000	0.001	0.000
Cynanchum thesiodes	0.000	0.008	0.000	0.000	0.000
Orostachys fimbriata	0.000	0.008	0.000	0.000	0.000
Melilotus suaveolens	0.000	0.009	0.000	0.000	0.000
Potentilla acaulis	0.000	0.000	0.010	0.000	0.000
Achnatherum sibiricum	0.000	0.000	0.013	0.000	0.000
Artemisia frigida	0.000	0.008	0.021	0.001	0.031
Potentilla bifurca	0.000	0.000	0.000	0.000	0.010

Table S2 Correlations between vegetation variables and grazing intensities

Vegetation variables	Cover	Biomass	Height	Density
r	-0.799	-0.909	-0.942	-0.07617
P value	0.0004	<.0001	<.0001	0.7873

Table S3 Correlations between plant variables and insect groups

Variables		Formicidae	Acrididae	Cicadellidae	Primary consumer	Secondary consumer
Cover	*r*	0.30948	0.49879	0.65558	0.58716	0.5778
	P value	0.2617	0.0584	0.008	0.0214	0.0241
Biomass	*r*	0.02354	0.59694	0.68374	0.61999	0.671
	P value	0.9336	0.0188	0.0049	0.0137	0.0062
Height	*r*	0.12961	0.71274	0.72823	0.71564	0.71438
	P value	0.6453	0.0029	0.0021	0.0027	0.0028
Density	*r*	0.25251	0.31363	0.35154	0.25617	0.37534
	P value	0.3639	0.255	0.1988	0.3568	0.168

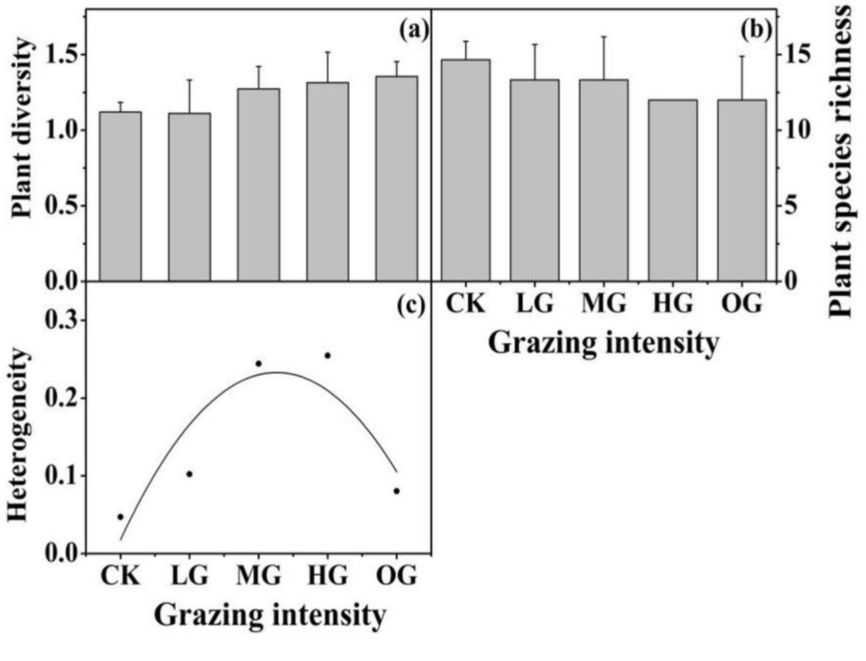

Fig. S1 Effects of grazing intensities on plant diversity, plant species richness and vegetation structure heterogeneity. Values represent means ± SE. Different lowercase letters above the bars indicate that values differ significantly between altered grazing intensities at $P < 0.05$.
